# Evaluation of the neutrophil‐to‐lymphocyte ratio, monocyte‐to‐lymphocyte ratio, and red cell distribution width for the prediction of prognosis of patients with hepatitis B virus‐related decompensated cirrhosis

**DOI:** 10.1002/jcla.23478

**Published:** 2020-07-14

**Authors:** XinKe Li, JianPing Wu, WeiLin Mao

**Affiliations:** ^1^ Department of Radiation Oncology College of Medicine The First Affiliated Hospital Zhejiang University Hangzhou China; ^2^ Department of Clinical Laboratory College of Medicine The First Affiliated Hospital Zhejiang University Hangzhou China

**Keywords:** decompensated cirrhosis, monocyte‐to‐lymphocyte ratio, mortality, neutrophil‐to‐lymphocyte ratio, red cell distribution width

## Abstract

**Background:**

The development and progression of hepatitis B virus‐related decompensated cirrhosis (DeCi) is associated with inflammatory responses. The monocyte‐to‐lymphocyte ratio (MLR), neutrophil‐to‐lymphocyte ratio (NLR), and red cell distribution width (RDW) are well‐known inflammation markers. We aimed to assess the utility of these parameters for predicating the prognosis of patients with HBV‐DeCi.

**Methods:**

We retrospectively recruited 174 patients diagnosed with HBV‐DeCi. Univariate and multivariate regression models were used to determine risk factors for mortality. Areas under the receiver operating characteristic curves were calculated to estimate and compare the predictive values of the three parameters. Hepatic function was evaluated using the Model for End‐Stage Liver Disease (MELD) score.

**Results:**

The NLR, RDW, and MLR were found to be significantly higher in patients who did not survive compared with surviving patients. Moreover, these variables were all able to predict early poor outcomes in patients with HBV‐DeCi, with NLR exhibiting the highest accuracy. Furthermore, a combination of the NLR and MELD score was a more accurate prognostic marker for predicting mortality than either marker alone in such patients.

**Conclusions:**

Hematological parameters can provide prognostic information for patients with HBV‐DeCi. Routine assessment of these parameters at admission may provide valuable data to complement other conventional measures for assessing disease condition in patients with HBV‐DeCi.

## Graphical abstract

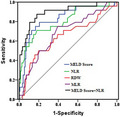

**Abbreviations**
ALT: Alanine aminotransferase;
AST: Aspartate aminotransferase;
AUCs: Areas under the curve;
BUN: Blood urea nitrogen;
CI: Confidence interval;
DeCi: Decompensated cirrhosis;
HBV: Hepatitis B virus;
HE: Hepatic encephalopathy;
HRS: Hepatorenal syndrome;
INR: International normalized ratio;
LC: Liver cirrhosis;
MELD score: Model for End‐stage liver disease score;
MLR: Monocyte‐to‐lymphocyte ratio;
NLR: Neutrophil‐to‐lymphocyte ratio;
RDW: Red cell distribution width;
ROC: Receiver operating characteristic;
SIRS: Systemic inflammatory response syndrome;
WBC: White blood cell

## 1 INTRODUCTION

Hepatitis B virus (HBV) infection is a major cause of liver cirrhosis (LC); 3% of cases of HBV‐compensated LC progress to decompensated cirrhosis (DeCi) each year in China.^1^, ^2^, ^3^ The condition of HBV‐related decompensated cirrhosis (HBV‐DeCi) is characterized by overt clinical features, which ultimately lead to death of the patient.^4^ The prognosis of DeCi is markedly worse, with median survival of 2‐4 years compared with 10‐12 years in compensated cirrhosis.^5^ Systemic inflammatory response syndrome (SIRS) is relatively common in patients with complicated cirrhosis and is increasingly recognized to play an important role in the development and progression of LC.^6^, ^7^ The neutrophil–to‐lymphocyte ratio (NLR), red cell distribution width (RDW), and monocyte‐lymphocyte ratio (MLR) are known to be inflammatory response markers^8^ which are easily evaluated from blood samples. Although these parameters have been investigated in patients with HBV‐DeCi,^9^, ^10^, ^11^, ^12^ there have been few studies evaluating all of these markers simultaneously in such patients. Therefore, the present study aimed to evaluate the roles of the RDW, NLR, and MLR in predicating the prognosis of patients with HBV‐DeCi. To the best of our knowledge, this is the first study to analyze these markers together.

## 2 MATERIALS AND METHODS

### 2.1 Patients

This study was performed according to the ethical guidelines of the Declaration of Helsinki and was approved by the Ethics Committee of the First Affiliated Hospital, School of Medicine, Zhejiang University.

We recruited all consecutive patients with HBV‐DeCi who were treated at our hospital from July 2017 to December 2019 for this retrospective study. Cirrhosis was diagnosed by histopathological examination or clinical laboratory tests and imaging studies. Liver decompensation was determined by the presence of various complications including ascites, hepatorenal syndrome (HRS), hepatic encephalopathy (HE), and/or variceal hemorrhage. In the present study, there were no exclusions for age/sex. The following exclusion criteria were applied: (a) presence of HAV, HCV, HIV, or other viral infection; (b) presence of chronic liver disease (eg, autoimmune hepatitis, alcoholic liver disease, or drug‐induced liver injury); (c) malignancy; (d) presence of any other blood system diseases; (e) cardiovascular diseases; and (f) received interferon, corticosteroid, or immunosuppressive therapy 6 months before admission were excluded. All participants received antiviral therapy from the start date.

### 2.2 Data extraction

For each patient, demographic and baseline clinical data (including age; sex; complications related to liver disease such as ascites, HE, and HRS; and clinical course in the hospital) were obtained from medical records and were recorded in a specific liver disease pro forma. Laboratory variables that were evaluated included levels of total protein, serum albumin, alanine aminotransferase (ALT), aspartate aminotransferase (AST), total bilirubin, creatinine, and blood urea nitrogen (BUN) as well as the international normalized ratio (INR). All biochemical values were measured using a Hitachi 7600 clinical analyzer (Hitachi) and a Sysmex CA1500 fully automatic analyzer (Sysmex Corp.). Hematological parameters including white blood cell (WBC) count and the relevant subpopulations (ie, lymphocyte, neutrophil, and monocyte counts), RDW, platelet counts, and hemoglobin levels were analyzed using an automated analyzer (Sysmex XN‐9000). The MLR and NLR were calculated by dividing the number of monocytes by lymphocytes and by dividing the neutrophil count by lymphocyte count, respectively. Additionally, hepatic function was evaluated using the Model for End‐Stage Liver Disease (MELD) score, which was calculated as previously described.^13^ The 28‐day patient survival rate was determined. Date of death was obtained from medical records.

### 2.3 Statistical analysis

All continuous variables are presented as mean ± standard deviation (mean ± SD) or median with interquartile ranges (IQR). Categorical data are presented as percentages. Comparisons between the non‐surviving and surviving groups were carried out using the Student's *t* test, the Mann‐Whitney *U* test, or the chi‐square test, as appropriate. Associations between variables were explored using Spearman's correlation analysis. To assess diagnostic value, logistic regression and receiver operating curve analyses (ROC) were performed. Statistical analyses were performed using Statistical Package for the Social Sciences (SPSS) 11.0 software (SPSS, Inc.) and MedCalc 12.0 software (Mariakerke, Belgium). We considered *P* < .05 to indicate statistical significance.

## 3 RESULTS

### 3.1 Patient characteristics

**Figure 1:**
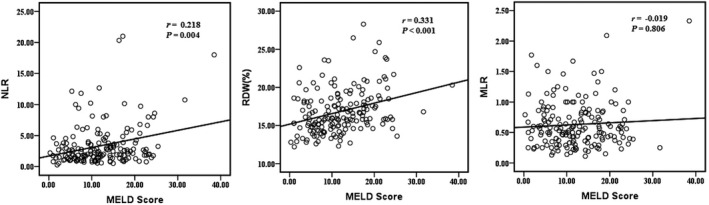
Scatter graphs showing correlations between the neutrophil‐to‐lymphocyte ratio, red cell distribution width, monocyte‐to‐lymphocyte ratio, and Model for End‐Stage Liver Disease scores in patients with hepatitis B virus‐related decompensated cirrhosis. MELD, Model for End‐Stage Liver Disease; MLR, monocyte‐to‐lymphocyte ratio; NLR, neutrophil‐to‐lymphocyte ratio; RDW, red cell distribution width

Of the 213 patients that were recruited for the present study, 39 were excluded: five due to hepatocellular carcinoma, two due to liver transplantation, four due to autoimmune liver disease, three due to HIV infection, 14 due to concurrent infection with hepatitis C/D/E/G, four due to alcoholic liver disease, two due to blood system diseases (iron deficiency anemia or chronic lymphocytic leukemia), and five due to undergoing immunomodulatory therapy (steroids or interferon therapy). Finally, 174 patients (139 males and 35 females) with HBV‐DeCi were enrolled, with a mean age of 53.6 ± 11.4 years. The most common complications were ascites in 114 patients (65.5%), followed by gastrointestinal bleeding in 50 patients (28.7%), HRS in 31 patients (17.8%), and HE in 4 patients (2.3%). Sixty‐one patients (35.1%) had more than one feature of decompensation at the time of first presentation. The median values of NLR, RDW, and MLR at enrollment were 2.39 (IQR, 1.43‐3.80), 16.1 (IQR, 14.9‐18.4), and 0.36 (IQR, 0.56‐0.82), respectively. Positive correlations were found between the MELD scores and the NLR (*r* = .218, *P* = .004) and RDW (*r* = .331, *P* < .001) (Figure 1). In contrast, the MLR was not found to be correlated with MELD score (*r* = −.019, *P* = .806).

Of the total study population, 150 patients survived and 24 died, giving a 28‐day mortality rate of 13.8%. The cause of death was hepatic failure in five patients, upper gastrointestinal bleeding in seven, HE in four, and HRS in eight. Demographic, clinical, and laboratory parameters of non‐survivors and survivors are shown in Table 1. There were no significant differences in age, gender, serum albumin, ALT, AST, platelet count, hemoglobin level, or monocyte count between the two groups. However, total bilirubin, creatinine, BUN, INR, WBC, neutrophil count, RDW, MLR, and MELD score were significantly higher in the non‐survivor group, while total protein level and lymphocyte count were significantly decreased among non‐survivors. Notably, the median NLR value was nearly 3‐fold higher among non‐survivors (2.00; IQR, 1.33‐3.33) compared with survivors (6.41; IQR, 2.61‐10.18; *P* < .001).

**Table 1:** Comparison of baseline characteristics of survivors and non‐survivors

	All patients (n = 174)	Non‐surviving patients (n = 24)	Surviving patients (n = 150)	*P*
Gender (female/male)	35/139	6/18	29/121	.712
Age (years)	53.6 ± 11.4	54.6 ± 11.4	53.4 ± 11.4	.634
Total protein (g/L)	61.5 ± 8.0	58.4 ± 10.3	62.0 ± 7.5	.040
Albumin (g/L)	30.9 ± 5.7	30.0 ± 5.4	31.0 ± 5.7	.409
ALT (U/L)	30.0 (17.0‐51.0)	38.5 (21.5‐59.0)	30.0 (17.0‐48.0)	.300
AST (U/L)	46.0 (28.0‐74.0)	51.5 (30.5‐113.5)	46.0 (28.0‐72.8)	.318
Serum creatinine (mmol/L)	73.0 (60.8‐87.0)	104.5 (62.5‐127.0)	72.0 (60.0‐84.0)	.006
Total bilirubin (μmol/L)	40.5 (18.0‐96.8)	84.0 (54.5‐249.0)	34.5 (17.0‐84.0)	.001
BUN (μmol/L)	5.70 (4.28‐7.60)	8.20 (6.00‐12.50)	5.50 (4.13‐7.28)	.001
INR	1.45 ± 0.38	1.74 ± 0.53	1.40 ± 0.33	<.001
WBC count (×10⁹/L)	4.1 (2.7‐5.5)	5.1 (4.2‐8.7)	4.0 (2.7‐5.3)	.005
Neutrophil count (×10⁹/L)	2.35 (1.48‐3.40)	3.75 (2.55‐7.20)	2.10 (1.40‐3.10)	.001
Lymphocyte count (×10^9^/L)	1.00 (0.70‐1.40)	0.75 (0.50‐1.10)	1.00 (0.70‐1.40)	.042
Monocyte count (×10⁹/L)	0.50 (0.30‐0.80)	0.60 (0.30‐1.00)	0.50 (0.30‐0.80)	.521
NLR	2.39 (1.43‐3.80)	6.41 (2.61‐10.18)	2.00 (1.33‐3.33)	<.001
MLR	0.36 (0.56‐0.82)	0.77 (0.55‐1.07)	0.50 (0.36‐0.75)	.004
RDW	16.1 (14.9‐18.4)	18.0 (15.5‐20.6)	15.9 (14.8‐18.0)	.025
Platelet count (×10⁹/L)	65.7 (43.0‐115.5)	68.5 (57.5‐134.5)	67.5 (43.0‐114.0)	.480
Hemoglobin (g/L)	103.5 ± 23.5	100.0 ± 21.2	104.1 ± 23.9	.438
MELD score	11.5 (6.8‐17.2)	20.2 (16.8‐22.5)	10.6 (6.2‐14.9)	<.001

Note

Data are expressed as n, mean ± standard deviation, or median (interquartile range).

Abbreviations: ALT, alanine aminotransferase; AST, aspartate aminotransferase; BUN, blood urea nitrogen; HBV‐DC, hepatitis B virus‐related‐decompensated cirrhosis INR, international normalized ratio; MELD score, Model for End‐Stage Liver Disease score; MLR, monocyte‐to‐lymphocyte ratio; NLR, neutrophil‐to‐lymphocyte ratio; RDW, red cell distribution width; WBC, white blood cell.

### 3.2 The utility of RDW, NLR, and MLR for predicting mortality in patients with HBV‐DeCi

**Figure 2:**
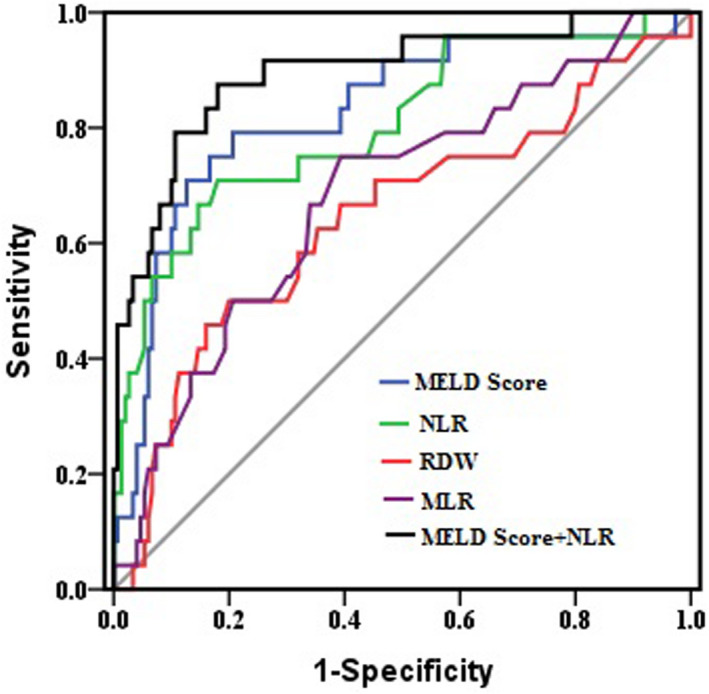
Receiver operating characteristic curve analysis by neutrophil‐to‐lymphocyte ratio, red cell distribution width, monocyte‐to‐lymphocyte ratio, Model for End‐Stage Liver Disease score, and neutrophil‐to‐lymphocyte ratio combined with Model for End‐Stage Liver Disease score for predicting mortality in patients with hepatitis B virus‐related decompensated cirrhosis. MELD, Model for End‐Stage Liver Disease; MLR, monocyte‐to‐lymphocyte ratio; NLR, neutrophil‐to‐lymphocyte ratio; RDW, red cell distribution width

Analysis of the associations between mortality and three parameters and MELD score by univariate logistic regression revealed that a higher MELD score, RDW, NLR, and MLR are associated with 28‐day mortality in patients with DeCi. Multivariate analysis revealed that only MELD and NLR remained independently associated with 28‐day mortality (Table 2). The results of ROC curve analysis (Figure 2) revealed the area under the curve (AUC) for NLR for predicting mortality to be 0.804, which was superior to both MLR (0.681) and RDW (0.643), but slightly inferior to MELD score (0.827) (Table 3). When NLR and MELD score were analyzed in combination, the AUC was 0.895—higher than that of NLR (Z = 1.650, *P* < .05) and MELD score (Z = 2.081, *P* < .05)—and the specificity (87.5%) the sensitivity (82.0%) improved.

**Table 2:** Results of multivariate analysis identifying independent factors associated with outcomes of patients with hepatitis B virus‐related decompensated cirrhosis

	Univariable hazard ratio	95% CI	*P*	Multivariable hazard ratio	95% CI	*P*
MELD score	1.237	1.132‐1.352	<.001	1.253	1.121‐1.397	<.001
NLR	1.371	1.194‐1.575	<.001	1.470	1.220‐1.772	<.001
MLR	4.172	1.521‐11.440	.006			
RDW	1.148	1.004‐1.313	.044			

Abbreviations: CI, confidence interval; MELD, Model for End‐Stage Liver Disease; MLR, monocyte‐to‐lymphocyte ratio; NLR, neutrophil‐to‐lymphocyte ratio; RDW, red cell distribution width.

**Table 3:** Predictive power of various scores for 28‐d mortality

	AUC (95% CI)	*P*	Cutoff value	Sensitivity	Specificity
MELD score	0.827 (0.762‐0.880)^a^	<.001	15.8	79.2	79.3
NLR	0.804 (0.737‐0.860)^b^	<.001	3.78	70.8	82.0
MLR	0.681 (0.607‐0.750)^c^	.003	0.59	75.0	60.7
RDW	0.643 (0.567‐0.714)^d^	.036	18.4	50.0	80.0

Note

a vs b: *P* = .770; a vs c: *P* = .094; a vs d: *P* = .001; b vs c: *P* = .028; b vs d: *P* = .049; b vs d: *P* = .661

Abbreviations: AUC, area under curve; CI, confidence interval; MELD, Model for End‐Stage Liver Disease; MLR, monocyte‐to‐lymphocyte ratio; NLR, neutrophil‐to‐lymphocyte ratio; RDW, red cell distribution width.

## 4 DISCUSSION

The role of the NLR, RDW, and MLR in patients with HBV‐DeCi has been extensively studied, but the present study is the first to compare prognostic capabilities of these parameters within a single study. Our results demonstrate that the NLR, RDW, and MLR were significantly higher among non‐surviving than surviving patients. More importantly, we also found that the NLR, RDW, and MLR were all able to predict early unfavorable outcomes in these patients, among which NLR was the most accurate for predicting mortality. Furthermore, the combination of NLR and MELD was found to be a more accurate prognostic index for the prediction of mortality than either marker alone in patients with HBV‐DeCi.

Systemic inflammation is known to play an important role in the disease progression of HBV‐DeCi. The NLR has been identified as a potent inflammatory marker, associated with diagnostic and prognostic properties in various clinical problems.^14^, ^15^, ^16^, ^17^ Recent evidence has emerged which indicates that elevated NLR is an independent predictor of poor prognosis in patients with DeCi and hepatocellular carcinoma.^18^, ^19^ The present study confirmed NLR as a prognostic factor by univariate and multivariate analyses; moreover, the AUC of NLR for predicting mortality was superior to both the MLR and RDW. Additionally, the NLR was found to be positively correlated with MELD score; increased NLR is closely associated with disease severity and liver damage in HBV‐DeCi, with consequent high mortality. We also found that non‐surviving patients displayed higher neutrophil counts and lower lymphocyte counts than the surviving group. A previous study has shown that lymphocytes and neutrophils, which participate in the pathogenesis of various diseases, both make important contributions to the WBC count and play a key role in the immune defense system of the body.^20^ The neutrophil count reflects the inflammatory state throughout the course of disease progression, while the lymphocyte count represents the outcome of regulated immunity.^21^ A high WBC suggests the presence of acute infection; among the study population, we found the WBC count to be higher in the non‐surviving group than the surviving group. Hence, we can assume that elevated NLR reflects the severity of acute systemic inflammation which occurs following primary injury and influences the prognosis of patients with HBV‐DeCi.

Similar to NLR, MLR is also known to be an inflammatory marker.^8^ However, in the present study, MLR was not found to be an independent predictor of mortality by multivariate analysis. The elevated levels of MLR that were observed in non‐surviving patients primarily resulted from the slightly increased number of monocytes and decreased number of lymphocytes compared with surviving patients. The inflammatory response can trigger the release of monocytes from bone marrow to the peripheral blood.^22^ Furthermore, a lower lymphocyte count can indicate malnutrition as well as inflammatory status. Therefore, the elevated MLR that we observed among non‐surviving patients may suggest ongoing inflammation, which could lead to the poor prognosis.

The RDW is an automated measure of the size variation of circulating red blood cells and therefore reflects the heterogeneity of these cells.^23^ It has been reported that the RDW is elevated in patients with HBV infection and is correlated with the severity of liver damage.^24^ In line with these findings, our study identified a significant increase in RDW among non‐surviving compared with surviving patients. Moreover, the RDW was positively correlated with MELD score. Thus, RDW value may be associated with survival for patients with HBV‐DeCi. However, we did not determine RDW to be an independent predictor of 28‐day mortality from multivariate analysis, and its predictive ability was found to be inferior compared with the other two parameters. It is maybe that an increase in RDW is related to the complex pathogenesis of HBV‐DeCi.

The MELD score has been widely used as for organ allocation in liver transplantation and is the current standard prognostic tool for predicting the 3‐ to 6‐month survival of patients with liver failure.^13^ However, this scoring system is not suitable for approximately 15%‐20% of candidates for liver transplantation, because important factors such as HE, HRS, or inflammation, which can affect diagnoses, are not taken into consideration for the determination of MELD scores.^25^ In the present study, we compared the predictive abilities of the NLR, RDW, and MLR with the MELD score, and the MELD score showed better predictive power than the other three parameters. However, analysis of the NLR, RDW, and MLR requires only one or two blood samples to be tested and is therefore more economical and readily available than calculating the MELD score. A combination of the NLR and MELD would be a more accurate prognostic biomarker for predicting mortality than either marker alone in patients with HBV‐DeCi.

The present study has some limitations which warrant consideration. First, the retrospective study design may have led to selection bias. Second, we were unable to evaluate some inflammatory markers, such as C‐reactive protein or IL‐6, which may be helpful in establishing the mechanism underlying the findings presented here. Further verification in a multi‐center, prospective study is warranted.

In conclusion, we assessed the role of the NLR, RDW, and MLR in predicating poor prognosis of patients with HBV‐DeCi. We show that these parameters are useful for predicating the 28‐day mortality in such patients and that using a combination of the NLR and MELD score is the most accurate approach. According to our data, routine assessment of these parameters at the time of admission may provide valuable supplementary information to other conventional approaches for assessing disease condition in these patients. Further prospective clinical trials are required to confirm the current findings.
